# Identification and in silico structural and functional analysis of a trypsin-like protease from shrimp *Macrobrachium carcinus*

**DOI:** 10.7717/peerj.9030

**Published:** 2020-04-23

**Authors:** José M. Viader-Salvadó, José Alberto Aguilar Briseño, Juan A. Gallegos-López, José A. Fuentes-Garibay, Carlos Alfonso Alvarez-González, Martha Guerrero-Olazarán

**Affiliations:** 1 Instituto de Biotecnología, Facultad de Ciencias Biológicas, Universidad Autónoma de Nuevo León, San Nicolás de los Garza, Nuevo León, Mexico; 2 Laboratorio de Acuicultura Tropical, División Académica de Ciencias Biológicas, Universidad Juárez Autónoma de Tabasco, Villahermosa, Tabasco, Mexico

**Keywords:** Brachyurins, *Macrobrachium carcinus*, Serine proteases, Threonine proteases, Trypsin-like protease

## Abstract

*Macrobrachium carcinus* (Linnaeus, 1758) is a species of freshwater shrimp widely distributed from Florida southwards to southern Brazil, including southeast of Mexico. In the present work, we identified a putative trypsin-like protease cDNA fragment of 736 nucleotides from *M. carcinus* hepatopancreas tissue by the 3′RACE technique and compared the deduced amino acid sequence to other trypsin-related proteases to describe its structure and function relationship. The bioinformatics analyses showed that the deduced amino acid sequence likely corresponds to a trypsin-like protease closely related to brachyurins, which comprise a subset of serine proteases with collagenolytic activity found in crabs and other crustacea. The *M. carcinus* trypsin-like protease sequence showed a global sequence identity of 94% with an unpublished trypsin from *Macrobrachium rosenbergii* (GenBank accession no. AMQ98968), and only 57% with *Penaeus vannamei* trypsin (GenBank accession no. CAA60129). A detailed analysis of the amino acid sequence revealed specific differences with crustacean trypsins, such as the sequence motif at the beginning of the mature protein, activation mechanism of the corresponding zymogen, amino acid residues of the catalytic triad and residues responsible for substrate specificity.

## Introduction

In crustacea, the organ in the digestive tract called the hepatopancreas or midgut gland is known to have high proteolytic activity and it is responsible for assimilating ingested proteins. The proteolytic activity is mainly due to the presence of serine proteases, with trypsins being the main digestive endopeptidases (Muhlia-Almazán, Sánchez-Paz & García-Carreño, 2008; Linton et al., 2014; Perera et al., 2015). Trypsins (EC 3.4.21.4) are serine proteases found in the digestive systems of many vertebrates and invertebrates that cleave peptides at the carboxyl side of Lys or Arg residues. Because trypsins can cause tissue damage due to autologous protein hydrolysis, they are synthesized as inactive precursors (proenzymes or zymogens) called trypsinogens. The importance of trypsins in protein digestion is given by its role in the activation of other digestive proenzymes, and trypsinogen itself. The conversion of trypsinogen to active trypsin is initiated by the specific cleavage of a small N-terminal peptide by an autoactivation process or in mammals, by the action of enterokinase. In the shrimp *Penaeus vannamei*, trypsin synthesis from trypsinogen has been proposed from sequence analysis (Klein et al., 1996), the demonstration of trypsinogen storage in the midgut gland has been proved (Sainz et al., 2004), and recently we demonstrated a rapid trypsinogen autoactivation process (Guerrero-Olazarán et al., 2019).

Trypsin sequences from different species have been used as a model for studies of evolution. A continuous evolutionary divergence of trypsins from a common ancestor has been proposed (Rypniewski et al., 1994). Comparisons of activation peptide sequences have also been used to study the evolution and function of trypsinogen activation (Chen et al., 2003).

*Macrobrachium carcinus* (Linnaeus, 1758) is a species of freshwater shrimp widely distributed from Florida southwards to southern Brazil, including southeast of Mexico. This species has great aquaculture potential due to its large size, high fertility in captivity and resistance to handling and stress conditions. Furthermore, it has a short larval period, is omnivorous and its meat is of good quality and widely accepted. Another freshwater crustacean of interest to aquaculture, especially in Europe, is the crayfish *Astacus leptodactylus* that resembles a small lobster. Nevertheless, little is known about the digestive physiology of these species.

Rapid amplification of cDNA ends (RACE) is a widely used technique for obtaining a cDNA copy of a specific RNA transcript from a cell. In the 3′RACE version of this technique, cDNAs are synthesized in a reverse transcription reaction using an oligo-dT-adaptor primer directed to the natural polyA tail of eukaryotic mRNAs. In the second step, specific cDNA is amplified by a polymerase chain reaction (PCR) using a sense gene-specific primer and an anti-sense primer that is complementary to the adaptor sequence of the primer used in the first step (Frohman, Dush & Martin, 1988).

In this work, the cDNA sequence of a putative trypsin-like protease from *M. carcinus* hepatopancreas tissue was identified by the 3′RACE technique and the deduced amino acid sequence was compared to other trypsin-related proteases to describe structure and function relationship of the enzyme. Our findings contribute to the understanding of the digestive physiology of this species and the molecular mechanism of crustacean trypsins.

## Materials and Methods

### Specimens, plasmids, medium composition, chemicals and enzymes

*Macrobrachium carcinus* specimens were from the Grijalva River, Centla, Tabasco, Mexico (latitude 18°14′11.9″ N, longitude 92°39′49.4″). All oligonucleotides were purchased from Integrated (DNA Technologies, Inc., Coralville, IA, USA). *Escherichia coli* DH5α, pGEM-T easy vector, RQ1 RNase-free DNase, SV Total RNA Isolation System, and GoTaq DNA polymerase were purchased from Promega (Madison, WI, USA). Luria-Bertani (LB) agar plates (1% tryptone, 0.5% yeast extract, 1% NaCl, 15 g/L agar, pH 7.0) with 100 µg/mL ampicillin was used for *E. coli* transformants selection. *Pfu*Ultra II Fusion HotStart DNA polymerase and AccuScript High-Fidelity Reverse Transcriptase were from Agilent Technologies (Santa Clara, CA, USA). RNAlater was from Life Technologies (Gaithersburg, MD, USA). All chemicals were of analytical grade and purchased from Sigma–Aldrich Co. (St. Louis, MO, USA) or from Productos Químicos Monterrey (Monterrey, Nuevo León, Mexico).

### 3′RACE assay

Total RNA from *M. carcinus* hepatopancreas tissue was isolated using SV Total RNA Isolation System, treated with RQ1 RNase-free DNase and used to synthesize cDNA by reverse transcription using 14.7 µM of T17AP primer (5′-GACTCGAGTCGACATCGAT_17_-3′) (Frohman, Dush & Martin, 1988) and 2 µl of AccuScript High-Fidelity Reverse Transcriptase in a final reaction volume of 20 µl, according to the manufacturer’s recommendations. The cDNA was amplified by PCR using the RACEAP primer (5′-GACTCGAGTCGACATCG-3′) (Frohman, Dush & Martin, 1988) and the consensus primer Pig1 (5′-CACTTCTGCGGCGCCTCCAT-3′) designed from a highly-conserved region of crustacean trypsin nucleotide sequences. The PCR was performed in a PCR Multigene Mini thermal cycler (Labnet International Inc., Edison, NJ, USA) in a 25 µl reaction volume containing 0.5 µM of each primer, 0.24 mM dNTP’s each, 1× buffer, 1.25 U *Pfu*Ultra II Fusion HotStart DNA polymerase, and 2 µl of primary cDNA, with a 40-cycle amplification program under the following conditions: 95 °C for 20 s, 60 °C for 20 s and 72 °C for 30 s, with a first denaturation step at 95 °C for 1 min and a final extension step at 72 °C for 3 min.

### Cloning and sequencing

The amplified cDNAs were adenylated and cloned into the pGEM-T easy vector according to the manufacturer’s instructions and using *E. coli* DH5α as the host strain. Transformants were selected for their ability to grow on LB-agar plates with 100 µg/mL ampicillin at 37 °C. Colonies were then randomly selected and plasmids were isolated by alkaline lysis extraction. The presence of *M. carcinus* cDNAs in the plasmids was confirmed by PCR using the Pig1 and RACEAP primers, as described above for cDNA amplification but using GoTaq DNA polymerase. Positive plasmids, as determined by PCR analysis from the different colonies of *E. coli*, were sequenced using T7 and SP6 universal primers and an ABI Prism 310 sequencer (Applied Biosystems, Foster City, CA, USA) at the Molecular Biology Unit, Institute of Cellular Physiology, Universidad Nacional Autónoma de México. The sequences obtained with the T7 and SP6 primers were aligned using the Contig Assembly Program (CAP) module from the BioEdit 7.2.6 program (Hall, 1999). The consensus sequences generated by CAP were compared to sequences reported in the databases (non-redundant GenBank coding sequence translations, Protein Data Bank, SwissProt, Protein Information Resource and Protein Research Foundation excluding environmental samples from whole genome shotgun projects) using the Blastx tool (Gish & States, 1993; Altschul et al., 1997) of the National Center for Biotechnology Information (Bethesda, MD, USA).

### Bioinformatics analysis of *M. carcinus* trypsin-like protease

The putative signal peptide cleavage site prediction was performed with the SignalP 4.0 Server (Petersen et al., 2011). The deduced amino acid sequence was compared to the Pfam protein families database (Finn et al., 2014) and the Prosite database of annotated motif descriptors (Sigrist et al., 2013) for functional domain analysis, analyzed in the CATH database (Orengo et al., 1997; Dawson et al., 2017) for protein structure classification and in the MEROPS database (Rawlings, Barrett & Bateman, 2010) for classifying in a peptidase family. The protein model was constructed by protein threading using the Phyre2 server (Kelley et al., 2015) and visualized using the Swiss-PdbViewer/DeepView 4.1 (Guex & Peitsch, 1997), which was also used to perform a structural superposition of the protein model with the crayfish trypsin structure (PDB code: 2F91), and to determine residues that are more than 30% surface accessible. Multiple sequences were aligned with the Clustal Omega program (Sievers et al., 2011), which was also used to construct the dendrogram plot by the neighbor-joining method. The unrooted radial tree was visualized with MEGA 7 (Kumar, Stecher & Tamura, 2016). Potential glycosylation sites were predicted with the Glycosylation Predictor server (Hamby & Hirst, 2008). Unless otherwise stated, the percentages of sequence identity were calculated by the global Needleman–Wunsch pairwise alignments using Blast tools or by multiple sequence alignments using the Clustal Omega program. The residue position identification in the *M. carcinus* trypsin-like (Mc-TryL) protease is denoted according to the first amino acid residue of the preproenzyme. Thus, the first residue of the mature Mc-TryL protease started at position 31. The chymotrypsin-based conventional numbering system (chymo#) is also given for reference (Hartley, 1970).

## Results

### 3′RACE assay

The 3′RACE assay for RNA from *M. carcinus* hepatopancreas rendered mainly an 800-bp band on agarose gels. PCR analysis of the isolated pGEM-T plasmids from different colonies showed six products of 800, 754, 500, 345, 305 and 200 bp. After sequencing, all products showed the sequence of the Pig1 and RACEAP primers, however, only the 800-bp product harbored a poly(A) tail.

### Bioinformatics analysis of *M. carcinus* trypsin-like protease

The longer cDNA with the poly(A) tail obtained by sequencing consisted of 736 nucleotides (GenBank accession no. MH900228). Blastx analysis showed an ORF of 627 nucleotides that encodes for 208 amino acid residues with a sequence identity of 94% with an unpublished trypsin from the Malaysian shrimp *Macrobrachium rosenbergii* (GenBank accession no. AMQ98968) and 57% sequence identity with Pacific white shrimp (*Penaeus vannamei*) trypsin (GenBank accession no. CAA60129). The sequenced cDNA was thus assigned to a nucleotide sequence that encodes for a trypsin-like protease fragment of *M. carcinus*. A more detailed analysis showed that the sequenced cDNA fragment had an untranslated region (UTR) at the 3′ end of 91 nucleotides while the same region in *M. rosenbergii* has only 64 nucleotides. The 27 extra nucleotides of the *M. carcinus* 3′UTR shared no identity at all with the *M. rosenbergii* 3′UTR.

**Figure 1:**
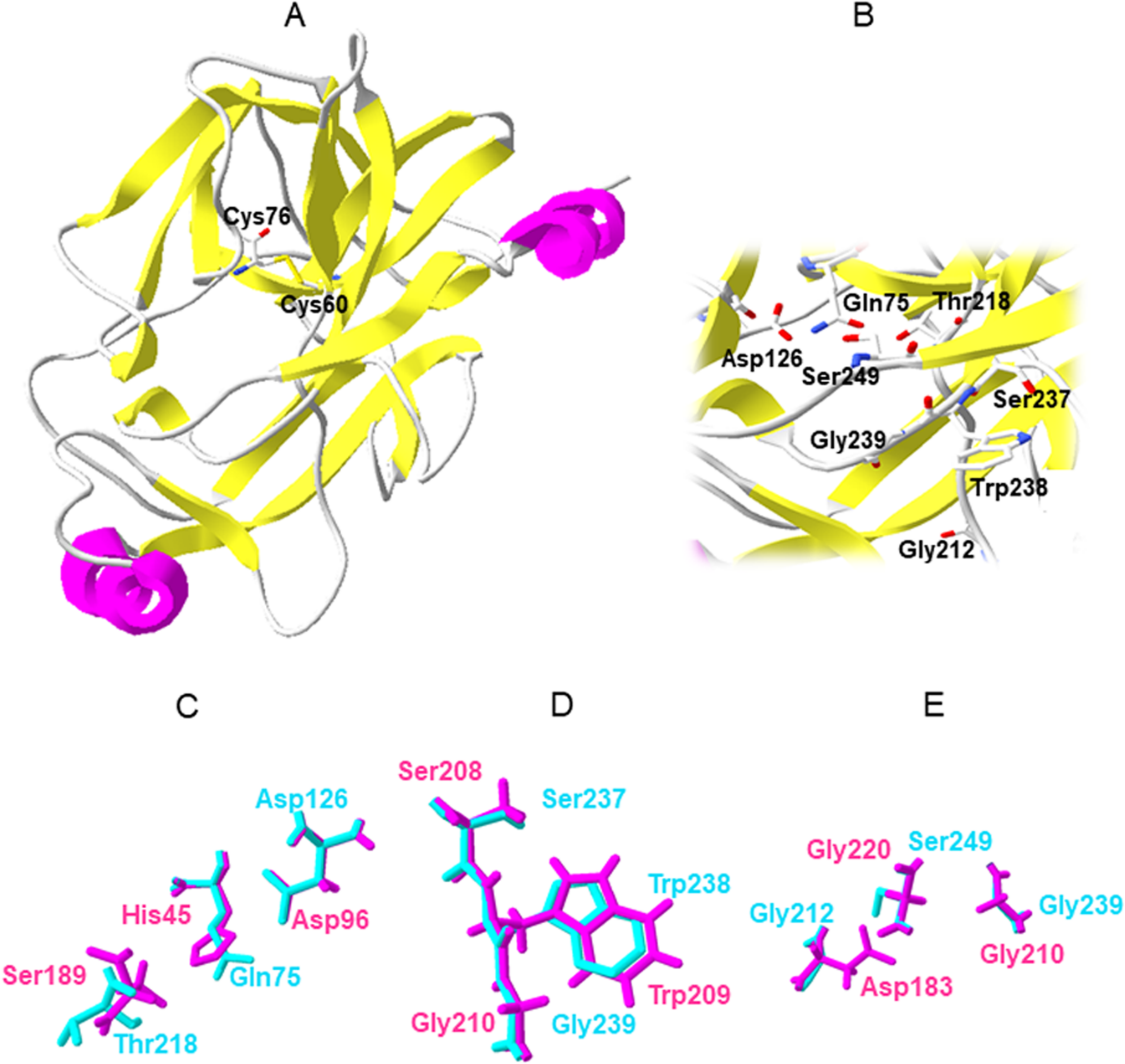
Molecular model of the Mc-TryL protease, and specific amino acid superposition of the Mc-TryL protease (cyan) and *A. leptodactylus* trypsin (magenta). (A) Molecular model of the Mc-TryL protease showing secondary structure elements and the disulfide bond Cys60–Cys76. (B) Enlarged view of the active site. (C) Catalytic triad. (D) Substrate binding residues. (E) Residues that confer the peptide-bond specificity. The numbers for Mc-TryL protease are according to the first amino acid residue of the preproenzyme, while for *A. leptodactylus* trypsin are based on mature protein.

Since the deduced amino acid sequence had height identity with the *M. rosenbergii* trypsin (94%), we continued the trypsin-like protease sequence analysis with a hybrid sequence; that is, 16 amino acid residues corresponding to the signal peptide (predicted by the SignalP 4.0 server), 14 amino acid residues corresponding to the propeptide and the first 28 amino acid residues of mature trypsin, all from the *M. rosenbergii* trypsin sequence. This was followed by the 208 amino acid residues from the trypsin-like protease sequence identified from *M. carcinus*. The first 28 amino acid residues from mature *M. rosenbergii* trypsin would likely differ in no more than 2 residues from the first residues sequence of Mc-TryL protease. According to the Pfam protein families database, the Mc-TryL protease contains a functional domain from residue 31 to 261 of the Trypsin PF00089 family. Other proteases, including trypsins, belong to this protein family. In addition, the CATH protein structure classification database classifies *M. carcinus* trypsin as 2.40.10.10 that corresponds to a mainly beta structure with a beta-barrel architecture, and thrombin topology, belonging to the serine protease homologous superfamily. The Phyre2 server constructed a full molecular model of the mature Mc-TryL protease (Fig. 1A) using the d2f91a1 fold from the 2F91 structure, corresponding to the *A. leptodactylus* trypsin (Fodor et al., 2005). This trypsin has a 53% sequence identity with the Mc-TryL protease and Phyre2 considered as a true homolog of Mc-TryL protease with 100% confidence. The Mc-TryL protease model showed a typical trypsin fold with 14 beta-strands and 2 alpha-helixes, covering 39% and 8% of the protein, respectively, and with the characteristic four loops that have been described for crayfish trypsin (Fodor et al., 2005).

**Figure 2:**
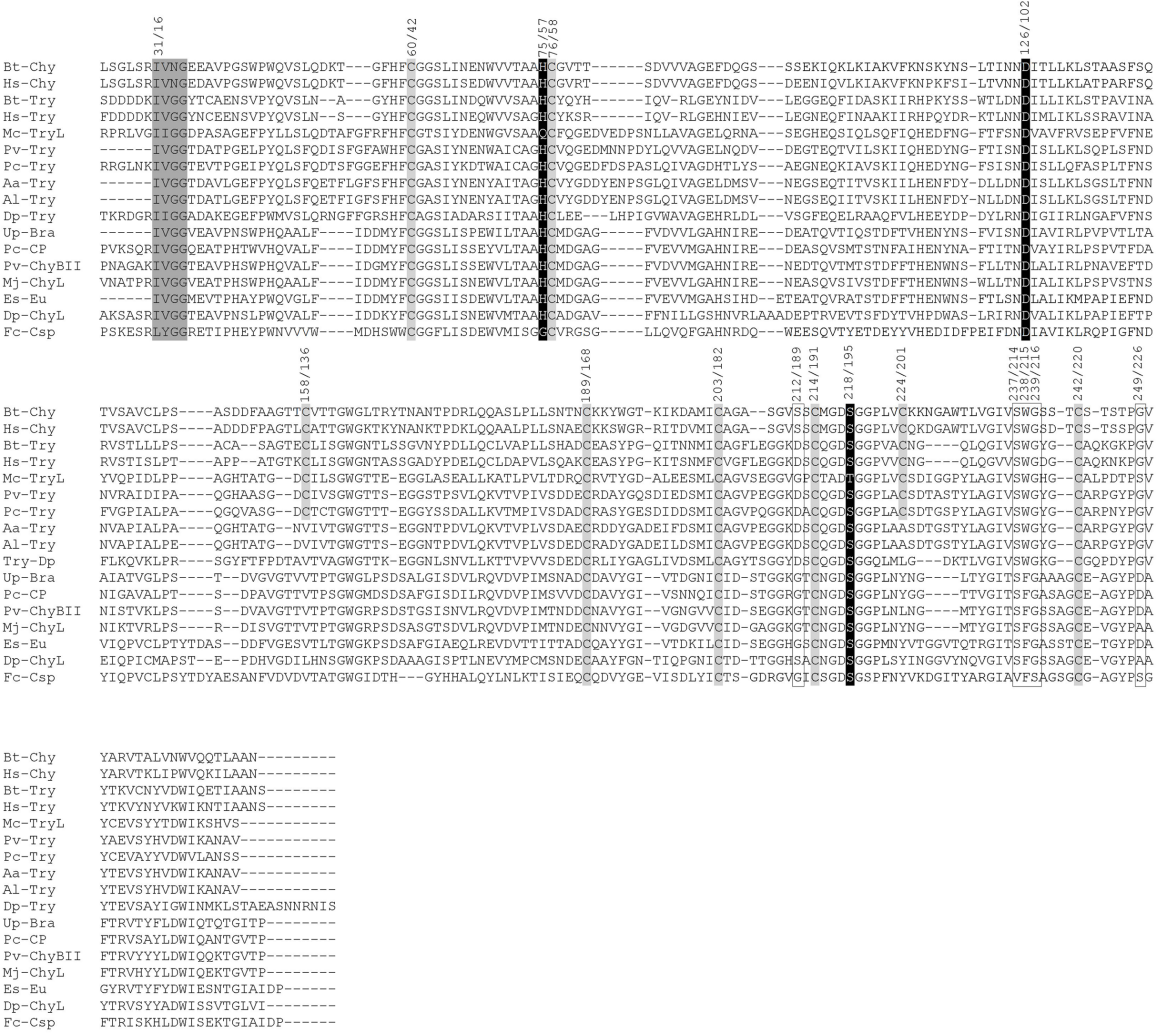
Multiple sequence alignment of the trypsin-like protease from *M. carcinus* (Mc-TryL), 12 mature brachyurins and 2 mammalian trypsins and 2 mammalian chymotrypsins as the reference. Grey shadows show sequence motif at the beginning of the mature protein, or conserved cysteines. The catalytic triad is denoted by black boxes, while substrate binding residues and residues that confer the peptide-bond specificity are shown by white boxes. The numbers above the sequences correspond to Mc-TryL protease position according to the first amino acid residue of the preproenzyme and second numbers are the chymotrypsin-based conventional numbering system. Five serine proteases classified by the MEROPS database as brachyurin-T: Pv-Try, trypsin from the Pacific white shrimp *Penaeus vannamei* (CAA60129); Pc-Try, trypsin from the red king crab *Paralithodes camtschaticus* (AAL67442); Aa-Try, trypsin from the noble crayfish *Astacus astacus* (P00765); Al-Try, trypsin from the narrow-clawed crayfish *Astacus leptodactylus* (Protein Data Bank (PDB) code 2F91); Dp-Try, trypsin from the water flea *Daphnia pulex* (EFX75427). Four serine proteases classified by the MEROPS database as brachyurin-C: Up-Bra, brachyurin (collagenolytic protease) from the Atlantic sand fiddler crab *Uca pugilator* (P00771), Pc-CP, collagenolytic serine protease from the red king crab *Paralithodes camtschaticus* (AAL67441), Pv-ChyBII, chymotrypsin BII from the Pacific white shrimp *Penaeus vannamei* (CAA71673), Mj-ChyL, chymotrypsin-like proteinase from the Japanese tiger prawn *Marsupenaeus japonicus* (BAI49929). Three serine proteases classified by the MEROPS database as euphauserase: Es-Eu, euphauserase from the antarctic krill *Euphausia superba* (MEROPS number MER0097318); Dp-ChyL, chymotrypsin-like protein from the water flea *Daphnia pulex* (EFX79603); Fc-Csp, collagenolytic serine protease from the Chinese white shrimp *Fenneropenaeus chinensis* (ACV97157). Mammalian trypsins and chymotrypsins: Hs-Try, trypsin from *Homo sapiens* (P07477); Bt-Try, trypsin from cattle *Bos taurus* (Q29463); Hs-Chy, chymotrypsin from *Homo sapiens* (P17538); Bt-Chy, chymotrypsin from cattle *Bos taurus* (P00766). Unless stated, GenBank accession numbers are given in parentheses.

The Prosite protein domain database and comparisons with other trypsins (Fig. 2) predicted four disulfide bonds for the Mc-TryL protease at Cys60 and Cys76, Cys158 and Cys224, Cys189 and Cys203 and Cys214 and Cys242. Nevertheless, only Cys60 and Cys76 remain close enough to form a disulfide bond according to the Mc-TryL protease molecular model and its superposition with the 2F91 structure; therefore, the Mc-TryL protease structure probably has only one disulfide bond (Fig. 1A). The Glycosylation Predictor server identified 14 potential glycosylation sites (2 N-linked and 12 O-linked glycosylations), 8 of which were likely to be glycosylated since they are at the protein surface. None of these are close to the active site. The MEROPS peptidases database classifies Mc-TryL protease in the Clan PA, Subclan PA(S), Peptidase family S1 (chymotrypsin family), Subfamily A, and Subfamily type S01.035, with the holotype brachyurin-T from *Astacus astacus* in a similar manner than crustacean trypsins, but not like mammalian trypsins that are classified in the Subfamily type S01.127.

A further analysis of the amino acid sequence revealed the absence of some typical trypsin sequence motifs. Most trypsin sequences, including crustacean trypsins, start with the IVGG motif, whereas the Mc-TryL protease sequence starts with the IIGG motif. In addition, the multiple sequence alignment analysis (Fig. 2) showed that for most trypsin sequences, the last amino acid residue of the propeptide is a Lys or Arg, which corresponds to the recognition site for trypsins, so that trypsins are involved in the activation process from trypsinogen to trypsin (Rypniewski et al., 1994). Nevertheless, Mc-TryL protease contains a Gly at the end of the propeptide and an Arg at the −4 position from the trypsin N-terminus. Therefore, the Mc-TryL protease cannot activate its zymogen in the same form like most other trypsins. The multiple sequence alignment (Fig. 2), Prosite analysis and molecular model showed that at the site of the typical catalytic triad Ser/His/Asp of serine proteases, the Mc-TryL protease contains Thr/Gln/Asp triad (218/75/126) (Figs. 1B and 1C). In addition, the multiple sequence alignment showed that the substrate binding residues are Ser237, Trp238 and Gly239 (Figs. 1D and 2), and Gly212, Gly239 and Ser249 are residues that confer the peptide-bond specificity to the Mc-TryL protease (Figs. 1E and 2).

**Figure 3:**
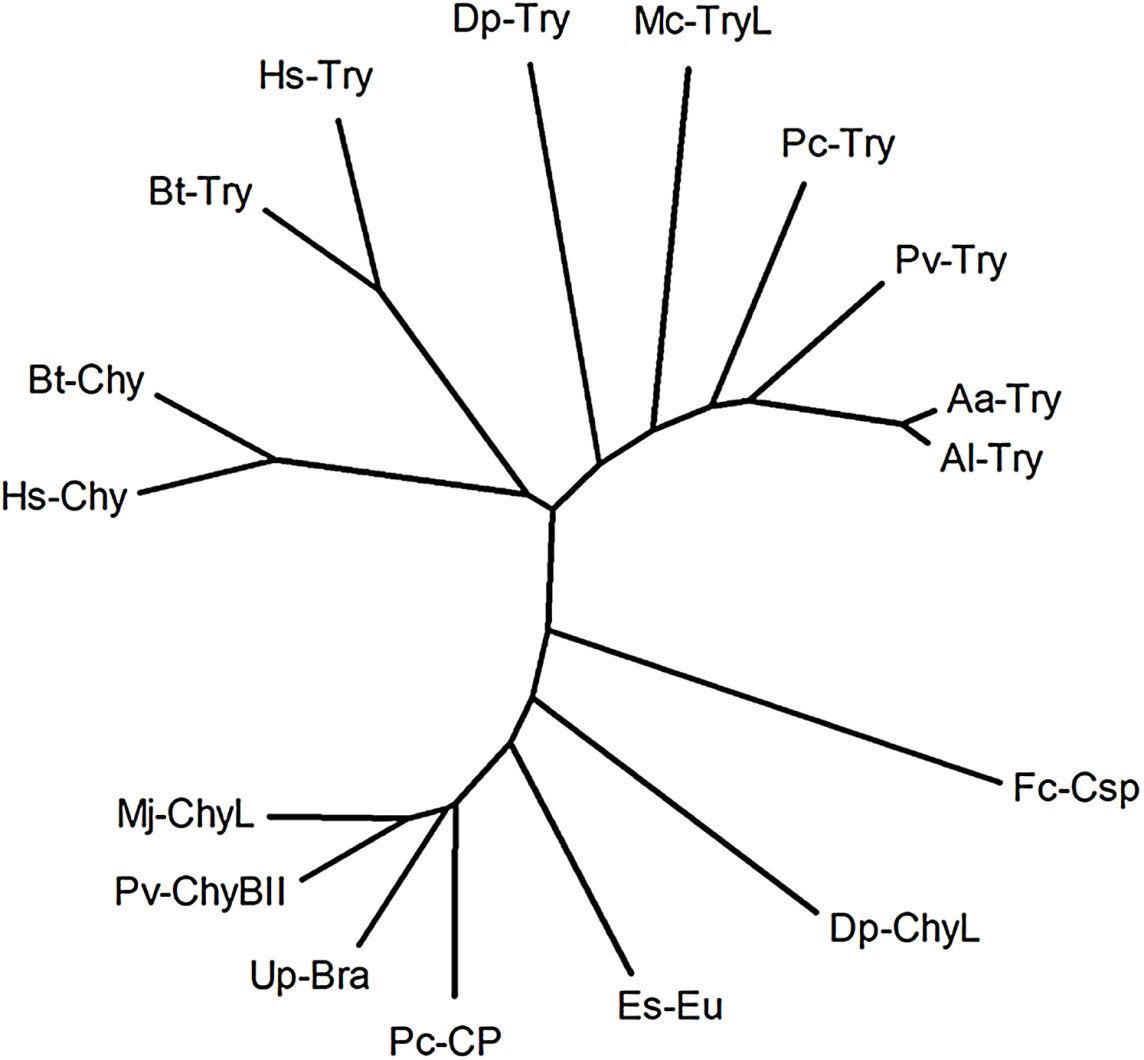
Neighbor-joining radial dendrogram from amino acid sequence alignment of the Mc-TryL protease, 12 mature brachyurins and two mammalian trypsins and two mammalian chymotrypsins as the reference. Abbreviations are as in Fig. 2.

Figure 3 shows a neighbor-joining radial dendrogram from the amino acid sequence alignment of 13 mature brachyurins, including the Mc-TryL protease and two mammalian trypsins and chymotrypsins as the reference. Every brachyurin-type protease forms a cluster, with the Mc-TryL protease being close to the brachyurin-T proteases, but only with a 57% sequence identity with the more related member of the cluster.

## Discussion

*Macrobrachium carcinus* is an endemic freshwater shrimp from southeast Mexico with potential use in aquaculture. Because proteases are the most important digestive enzymes in crustacea, in the present work we describe structural and functional implications of a Mc-TryL protease.

In our initial 3′RACE experiments, we used as the gene-specific primer a 5′-primer designed from a consensus sequence from the beginning of the crustacean trypsin coding sequences. Nevertheless, a sequence coding for a peptide with high identity to reported trypsins was not amplified. Instead, four sequences were amplified and after sequencing they showed a query coverage and local identity that was greater than 72% and 58%, respectively, with crustapain from the shrimp *Pandalus borealis* (GenBank accession no. AB091669). Crustapains are papain-like cysteine proteinases from crustacea species (Aoki, Ahsan & Watabe, 2003). For this reason, a new consensus 5′-primer (Pig1) was designed from crustacean trypsin sequences directed to a highly conserved internal region of the mature proteins. This new 3′RACE experiment led us to deduce an amino acid sequence of 208 residues with identity to the reported crustacean trypsins. Following our results, the trypsin sequence (cDNA and protein) of *M. rosenbergii* was reported at the GenBank database. This sequence explained why our initial 3′RACE design for fishing the *M. carcinus* trypsin mRNA failed, since the 5′ end of the *M. rosenbergii* trypsin cDNA has only 57% identity with the *P. vannamei* trypsin cDNA. Furthermore, the 3′ end of the consensus primer from the beginning of the crustacean trypsin coding sequences lacked complementarity at the position that would hybridize to *M. rosenbergii* trypsin cDNA sequence. With the amino acid sequence (208 residues) obtained in the present work and the *M. rosenbergii* trypsin sequence reported in the GenBank database, the most probable sequence of the Mc-TryL protease can be inferred.

The bioinformatics analyses showed that the deduced amino acid sequence likely corresponds to a trypsin-like protease that is closely related to brachyurins, which are a subset of serine proteases with collagenolytic activity found in crabs and other crustacea. These proteases possess a specificity that resembles a combination of trypsins, chymotrypsins and elastases, with activity (type Ia or brachyurin-C) or limited activity (type Ib or euphauserase) towards Arg substrates or with a strictly trypsin-like protease specificity (type II or brachyurins-T) (Rudenskaya, Kislitsin & Rebrikov, 2004; Page & Craik, 2013). The Mc-TryL protease sequence showed a global sequence identity of only 57% with the *P. vannamei* trypsin and a lower identity with other crustacean trypsins, which places it in the dendrogram somewhat separated from the group of crustacean trypsins. A detailed analysis of the amino acid sequence revealed specific differences from crustacean trypsins, such as the sequence motif at the beginning of the mature protein, activation mechanism of the corresponding zymogen, amino acid residues of the catalytic triad and residues responsible for substrate specificity.

Although most of the trypsin sequences start with the IVGG motif, including a low molecular weight trypsin from the hepatopancreas of *M. rosenbergii* (Sriket et al., 2012), the IIGG motif is also present in other trypsins, with the sequence of *Daphnia pulex* trypsin (GenBank accession no. EFX75427) being the most related to the Mc-TryL protease with the IIGG motif (44% sequence identity). After cleavage of the propeptide for trypsinogen activation, the N-terminus of trypsins is buried in the C-terminal domain forming an ion pair with Asp194 (chymo#) making several hydrogen bonds (Rypniewski et al., 1994). Since the V to I change is conservative, both N-terminal sequences (IVGG and IIGG) would likely have the same function. Furthermore, the multiple sequence alignment of Mc-TryL protease with other trypsin sequences (Fig. 2) showed that Mc-TryL protease contains Asp217, the equivalent position 194-chymo#.

As other crustacean trypsins, Mc-TryL protease does not contain a tetra-Asp sequence in the propeptide, neither Asp217 (chymo#), as containing other most vertebrate trypsinogens. This tetra-Asp sequence together with Asp217 (chymo#) has been involved in an inhibitory function for trypsin-mediated trypsinogen activation (autoactivation), by an electrostatic repulsion between Asp217 (chymo#) and the tetra-aspartate (Nemoda & Sahin-Tóth, 2007). Therefore, the corresponding trypsinogen of the Mc-TryL protease likely has an increased autoactivation rate in comparison with other non-crustacean trypsins in a similar manner as we have recently described for the *P. vannamei* trypsinogen (Guerrero-Olazarán et al., 2019), although with slight differences due to the lack of Arg or Lys at the end of the propeptide. Since the storage of active trypsin is a risk of tissue damage through hydrolysis of autologous protein, *M. carcinus* must possess a tightly regulated mechanism for controlling activation until needed for a precise function similar to other crustaceans. The control of trypsin activity in crustacea is a fine-tuning mechanism that involves the continuous regulation of trypsin activity through zymogen storage, secretion and activation like frequent feeder species (Sainz et al., 2004; Guerrero-Olazarán et al., 2019), with a main role of trypsin inhibitors for controlling trypsin activity and/or trypsinogen activation (García-Carreño, Carrillo & Navarrete del Toro, 1999; De Albuquerque-Cavalcanti, García-Carreño & Navarrete del Toro, 2002; Guerrero-Olazarán et al., 2019).

Other trypsins that are without an Arg or Lys at the end of the propeptide have already been described. For instance, *Musca domestica* (housefly) and *Aedes albopictus* (tiger mosquito) trypsins contain an Arg or Lys, respectively, at the −4 position from the trypsin N-terminus, like the Mc-TryL protease. For this type of propeptide, an activation mechanism through cleavage of the carboxy terminal of the Arg or Lys, followed by removal of some amino acid residues by an aminopeptidase has been initially suggested. Now, however, a propeptide cleavage by the action of a cathepsin has been proposed (Le et al., 2011). For the *M. carcinus* trypsin, the cathepsin involved in the zymogen activation could be cathepsin B since this protease mainly cleaves a peptide bond at the carboxy side of a Gly, if another Gly is located at the +4 position from the protease N-terminus (Rawlings, Barrett & Bateman, 2010), as happens with the Mc-TryL protease sequence.

Variations of the catalytic Ser/His/Asp triad configuration have been described for unconventional serine proteases (Ekici, Paetzel & Dalbey, 2008); however, the Mc-TryL protease contains Thr/Gln/Asp as the catalytic triad, which has not been previously described. Although Thr is as reactive as a nucleophile as Ser, Thr is rarely used in a similar functional context as is Ser. Nevertheless, a Ser195Thr (chymo#) substitution in human thrombin serine protease reduces but does not nullify activity (Pelc et al., 2015). This was explained by biases Thr mobility within the active site and stabilization of rotamers incompatible with substrate binding due to the additional methyl group on the side chain (Pelc et al., 2015). In any case, this substitution generated Thr/His/Asp triad still different from that of Mc-TryL protease, where His was changed by Gln, which has more mobility than His, furthermore, clash analysis by Phyre2 showed no clashes for Thr218. In the Mc-TryL protease catalytic triad, Thr would be the nucleophile, Gln would be the base catalyst through the imidic acid tautomer and Asp would help orient the Gln residue and be the base that catalyzes the Gln tautomerization via N-deprotonation. Amide-imidic acid tautomerization has been proposed for other enzyme mechanism (Nakamura et al., 2015; Grigorenko, Khrenova & Nemukhin, 2018). Thus, the sequence identified as the Mc-TryL protease is actually not a serine protease, but instead, it is a threonine protease. To the best of our knowledge, the sequence identified as the Mc-TryL protease is the first protease described with a trypsin domain with a Thr/Gln/Asp catalytic triad.

In serine proteases, the presence of Ser214, Trp215 and Gly216 (chymo#) are critical for efficient substrate hydrolysis (Hedstrom, 2002) since they form hydrogen bonds with P1 or P3 residues, Schechter and Berger nomenclature (Schechter & Berger, 1968). The Mc-TryL protease contains the same residues at equivalent positions (237, 238 and 239) ensuring the efficient protein binding of the substrate to the Mc-TryL protease.

The specificity of serine proteases is usually defined by the residues at positions 189, 216 and 226 (chymo#), which are in the S1 pocket, Schechter and Berger nomenclature (Schechter & Berger, 1968). This pocket is adjacent to Ser195 (chymo#) and is formed by residues 189–192, 214–216 and 224–228 (chymo#) (Hedstrom, 2002). For example, Ser189, Gly216 and Gly226 for chymotrypsins, Asp189, Gly216 and Gly226 for trypsins and Val190, Val216 and Thr226 for elastases (chymo#). In the case of the Mc-TryL protease, Gly, Gly, Ser are at these conserved positions (212, 239 and 249, respectively). Substitutions involving Gly189 (chymo#) have been described for chymotrypsin from the fire ant (*Solenopsis invicta*) (Botos et al., 2000), and for the collagenolytic protease from the hepatopancreas of crabs (*Uca pugilator* and *Paralithodes camtschaticus*) (Grant et al., 1980; Rudenskaya, Kislitsin & Rebrikov, 2004). Nevertheless, these proteases contain an Asp at position 226 (chymo#) resembling a 189 to 226 (chymo#) switch from the trypsin specificity site, while the Mc-TryL protease contains a Ser at this position, resembling a switch from the chymotrypsin specificity. The Gly189, Gly216 and Ser226 (chymo#) pattern has been previously described for unknown substrate specificity serine proteases by a bioinformatic analysis of putative serine proteases from the *Daphnia pulex* genome, the first crustacean genome that was sequenced (Julien, 2014).

The specific differences between the Mc-TryL protease sequence and crustacea trypsins approximate the Mc-TryL protease to crustacean collagenolytic proteases of type Ia and Ib brachyurins. The differences in the catalytic triad and specificity of residues likely confer a broad substrate specificity (including collagen) to the Mc-TryL protease. The ability to hydrolyze collagen reflects the diets of crustaceans, which include animal detritus that would contain a large amount of collagen. The global structure of trypsin (type II brachyurins) and the specific properties of type I brachyurins would place the Mc-TryL protease among these groups of proteases. Interestingly, *D. pulex* trypsin is also separated from the crustacea trypsins in the dendrogram.

Moreover, the high number of potential glycosylation sites, none of which close to the active site, could provide stability to the Mc-TryL protease, even stability from proteolysis by autolysis or by the action of other proteases since the oligosaccharides that are bound to this protease may be able to protect the proteolysis susceptible sites by steric hindrance.

## Conclusions

We identified a putative trypsin-like protease cDNA from *M. carcinus* hepatopancreas, deduced the amino acid sequence and described its structure and function relationship. The bioinformatics analyses showed a mainly beta structure for the Mc-TryL protease with a beta-barrel architecture and thrombin topology, belonging to the serine protease homologous superfamily. The amino acid sequence is closely related to brachyurins, but somewhat separated from other crustacean trypsins. The Mc-TryL protease sequence has important specific differences with this group, such as the sequence motif at the beginning of the mature protein, activation mechanism of the corresponding zymogen, amino acid residues of the catalytic triad and residues responsible for substrate specificity. Although eight serine proteases have recently been detected in the hepatopancreas of *M. carcinus* (Manríquez-Santos et al., 2018), the specificity of the Mc-TryL protease described in this work, which is also present in *M. rosenbergii*, may be broader than what is currently believed for other brachyurins. Therefore, further studies are needed to better understand the digestive physiology in this species.

## Supplemental Information

10.7717/peerj.9030/supp-1 Supplemental Information 1 Agarose gel (0.8% in TBE) of RT-PCR assays using AccuScript High-Fidelity Reverse Transcriptase, PfuUltra II Fusion HotStart DNA polymerase, T17AP primer for the RT step and Pig1 and RACEAP primers for the PCR step. Lane M, molecular size marker; lane 1, amplified product from *Macrobrachium carcinus* hepatopancreas RNA; lane 2, amplified product from *Penaeus vannamei* hepatopancreas RNA; lane 3, negative PCR control (amplification without target DNA).

10.7717/peerj.9030/supp-2 Supplemental Information 2 Agarose gels (0.8% in TBE) of PCR assays using GoTaq DNA polymerase and Pig1 and RACEAP primers. Lanes M, molecular size marker. Gel A, lane 5, negative PCR control; other lanes in both gels, amplified products from several pGEMPig DNAs isolated from different *E. coli* colonies.

10.7717/peerj.9030/supp-3 Supplemental Information 3 Agarose gels (0.8% in TBE) of PCR assays using GoTaq DNA polymerase and Pig1 and RACEAP primers. Lanes M, molecular size marker. A: lanes 1–6, amplified products from several pGEMPig DNAs isolated from different *E. coli* subcolonies from colony 45; lane 7, amplified product from *Macrobrachium carcinus* hepatopancreas RNA. B: lanes 1–6, amplified products from several pGEMPig DNAs isolated from different *E. coli* subcolonies from colony 46; lane 7, negative PCR control.

10.7717/peerj.9030/supp-4 Supplemental Information 4 T7 and SP6 sequences from pGEMPig452 and pGEMPig454 plasmids aligned using the Contig Assembly Program module from the BioEdit 7.2.6 program. Consensus sequence is also shown. Underline regions are the sequences of the Pig1 and RACEAP primers.

10.7717/peerj.9030/supp-5 Supplemental Information 5 Flow diagram of the methods used. *Macrobrachium carcinus* photo credit: Hans Hillewaert, licensed under the Creative Commons Attribution-Share Alike 4.0 International (https://commons.wikimedia.org/wiki/File:Macrobrachium_carcinus.jpg).

## Additional Information and Declarations

### DNA Deposition

The following information was supplied regarding the deposition of DNA sequences: Trypsin-like protease cDNA (MH900228) and amino acid sequences from *Macrobrachium carcinus* (AYN64566) are available at GenBank.

### Data Availability

The following information was supplied regarding data availability: The raw data is available at GenBank and in the Supplemental Files.
