# Supplementary figures and images for: Identification and in silico structural and functional analysis of a trypsin-like protease from shrimp Macrobrachium carcinus
Source: PeerJ. 2020 Apr 23;8:e9030. doi: 10.7717/peerj.9030 (PMC7183752; doi:10.7717/peerj.9030)

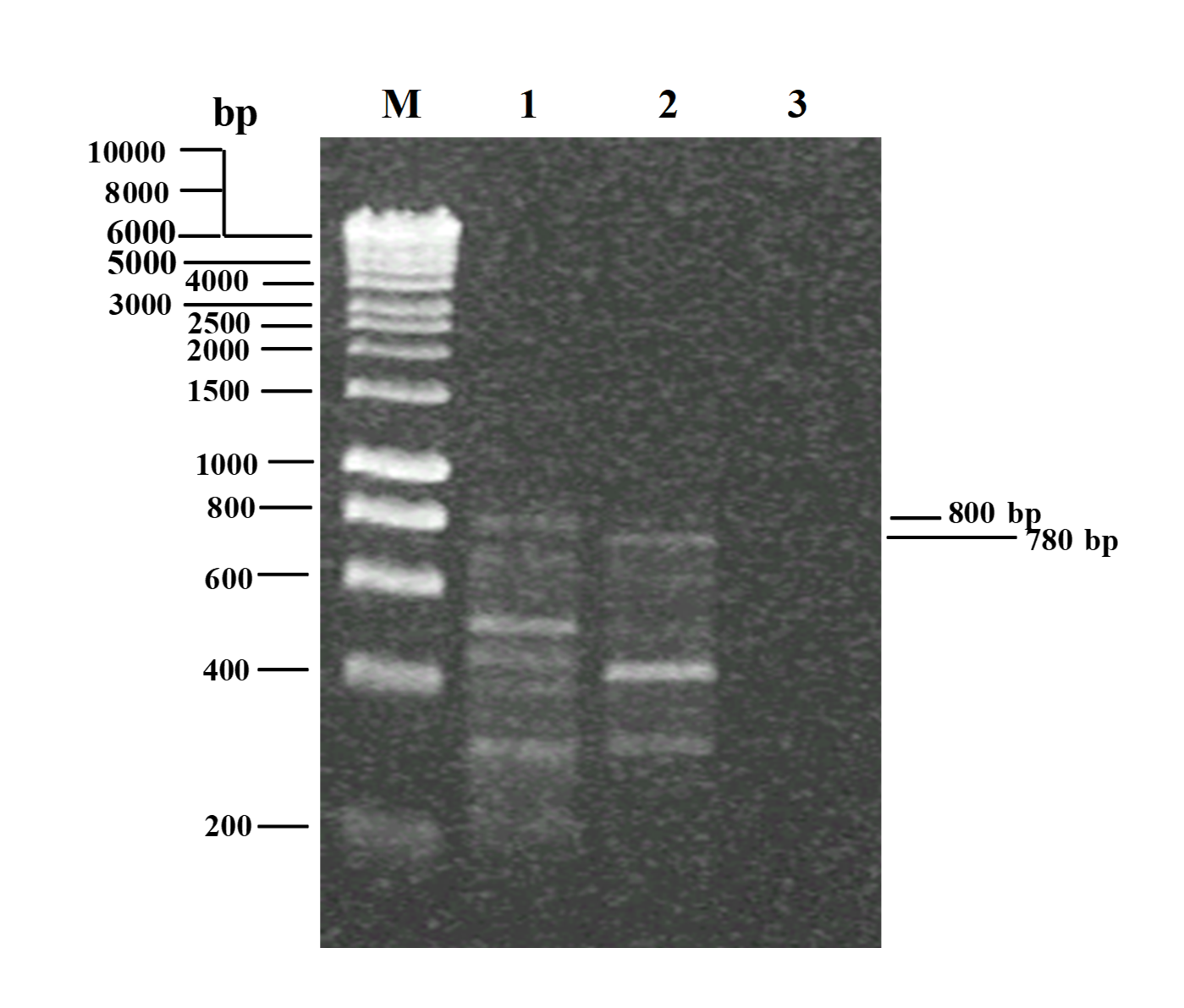

Supplement: Supplemental Information 1 — Lane M, molecular size marker; lane 1, amplified product from Macrobrachium carcinus hepatopancreas RNA; lane 2, amplified product from Penaeus vannamei hepatopancreas RNA; lane 3, negative PCR control (amplification without target DNA). [file peerj-08-9030-s001.png]

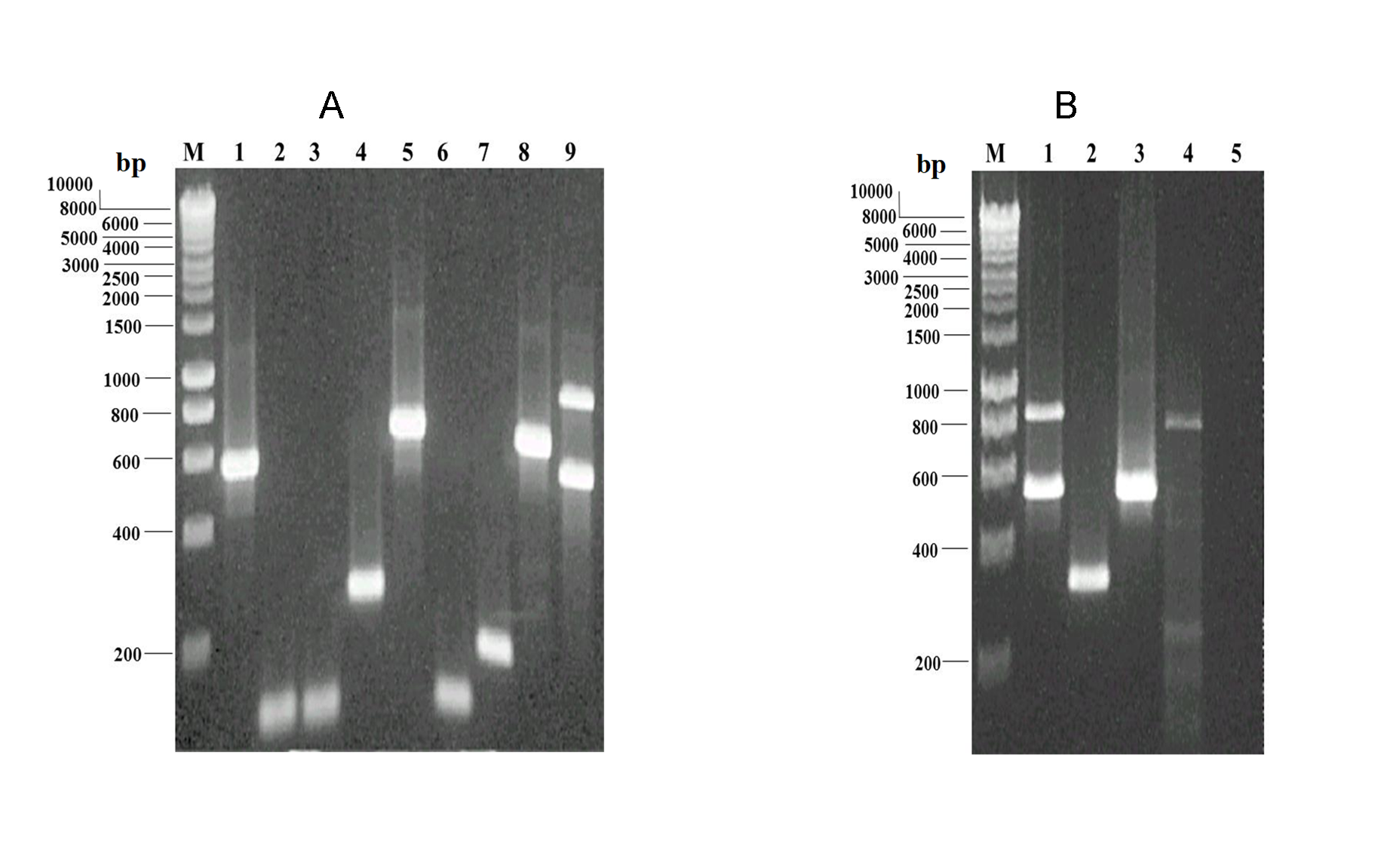

Supplement: Supplemental Information 2 — Lanes M, molecular size marker. Gel A, lane 5, negative PCR control; other lanes in both gels, amplified products from several pGEMPig DNAs isolated from different E. coli colonies. [file peerj-08-9030-s002.png]

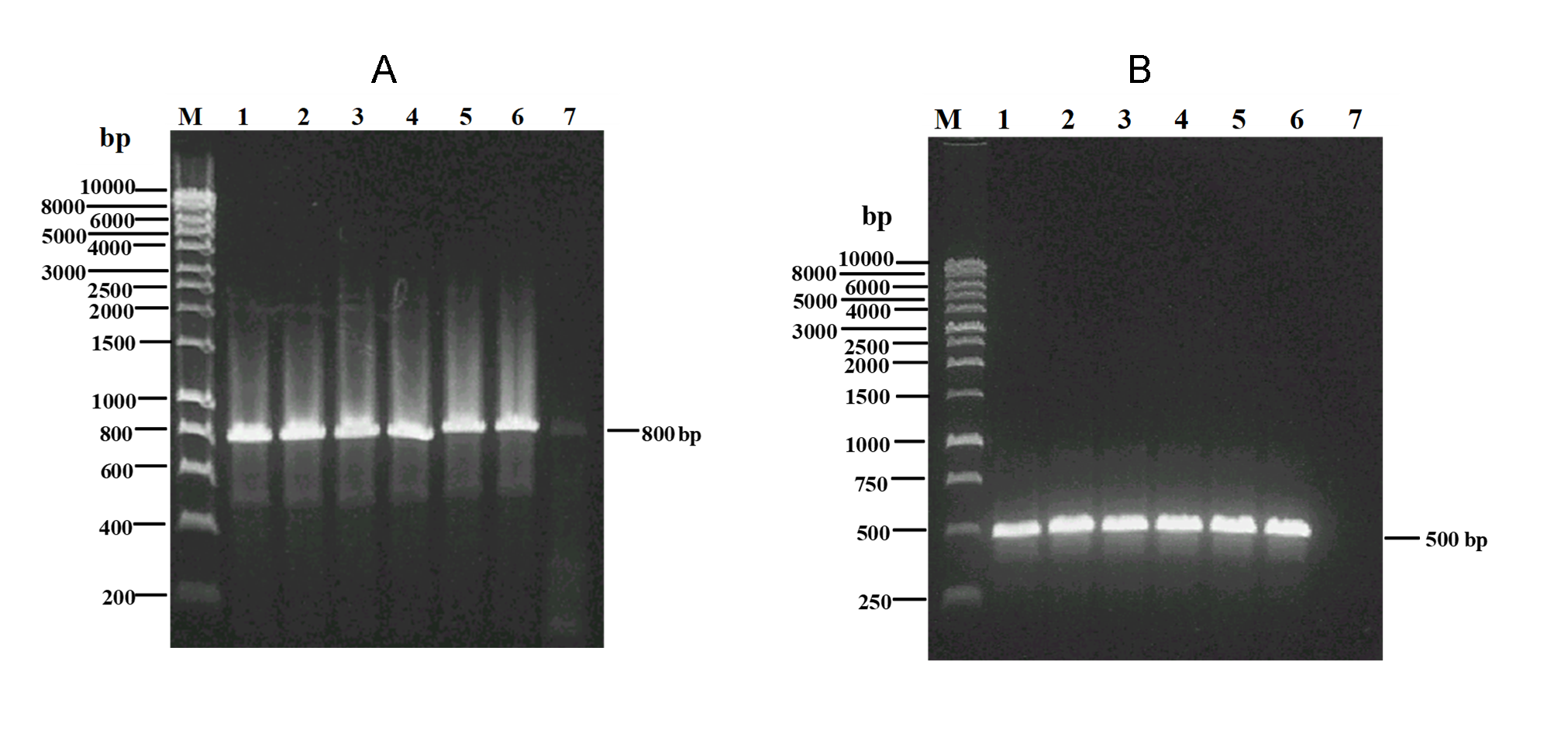

Supplement: Supplemental Information 3 — Lanes M, molecular size marker. A: lanes 1–6, amplified products from several pGEMPig DNAs isolated from different E. coli subcolonies from colony 45; lane 7, amplified product from Macrobrachium carcinus hepatopancreas RNA. B: lanes 1–6, amplified products from several pGEMPig DNAs isolated from different E. coli subcolonies from colony 46; lane 7, negative PCR control. [file peerj-08-9030-s003.png]

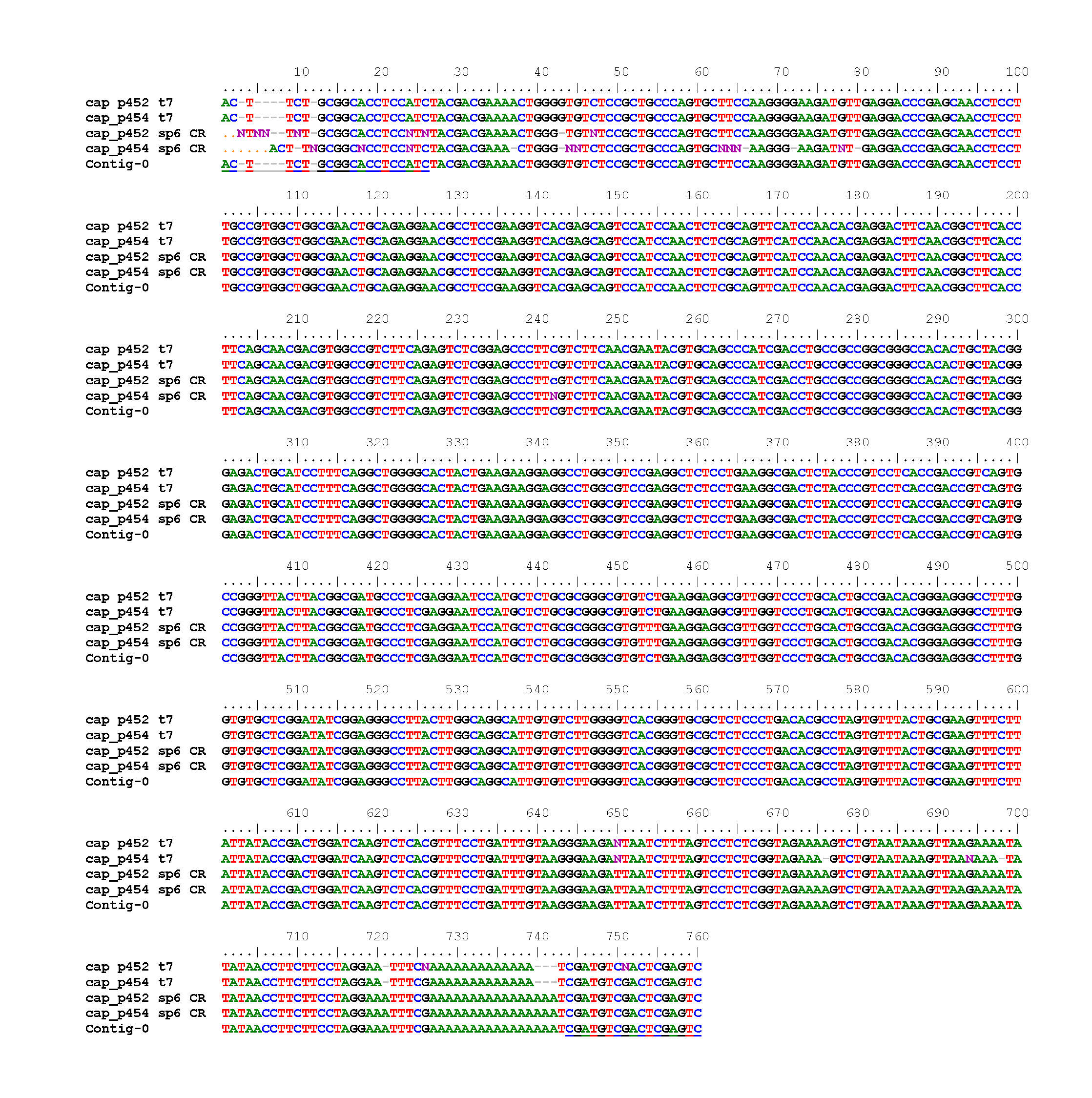

Supplement: Supplemental Information 4 — Consensus sequence is also shown. Underline regions are the sequences of the Pig1 and RACEAP primers. [file peerj-08-9030-s004.png]

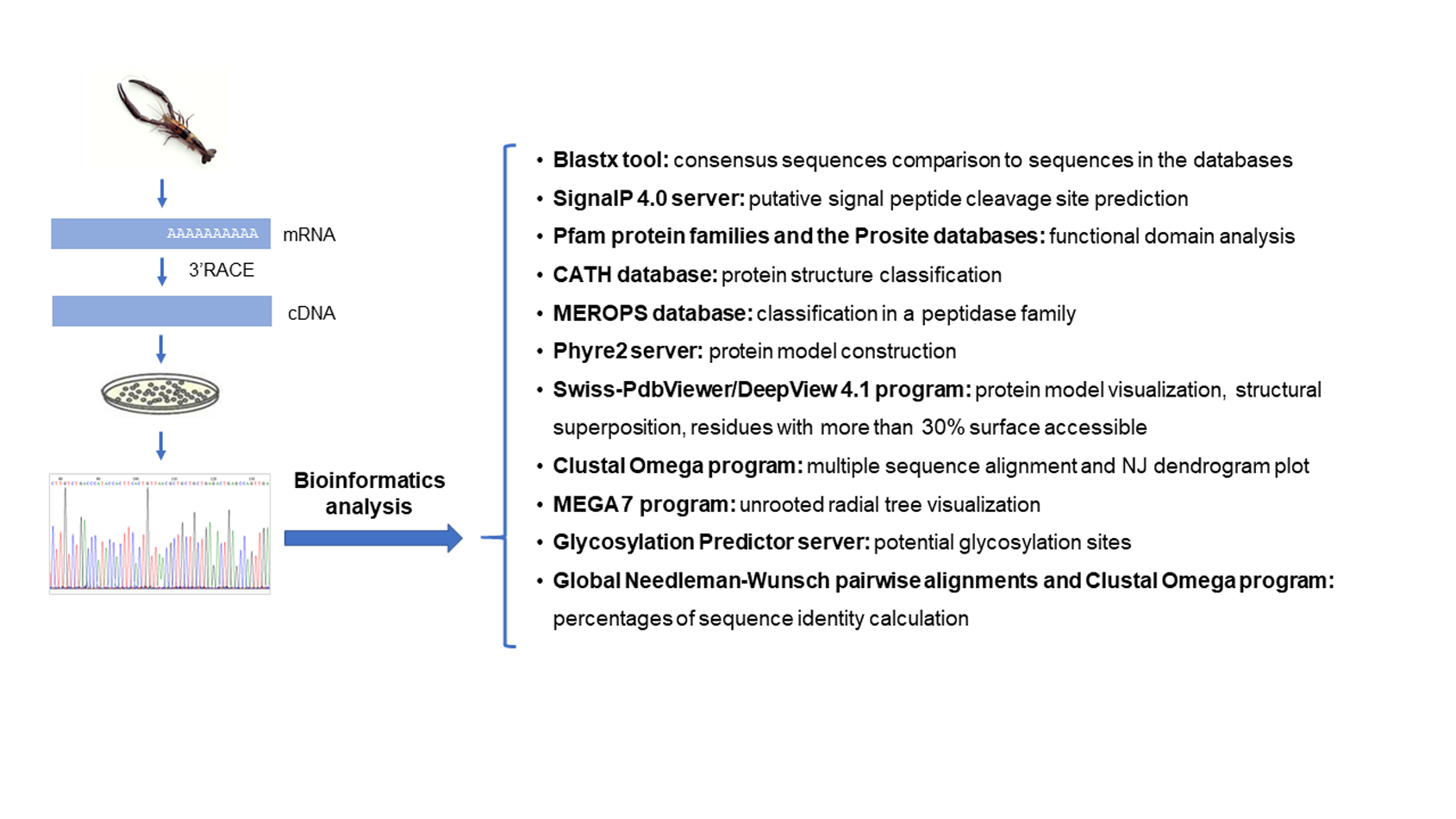

Supplement: Supplemental Information 5 — Macrobrachium carcinus photo credit: Hans Hillewaert, licensed under the Creative Commons Attribution-Share Alike 4.0 International (https://commons.wikimedia.org/wiki/File:Macrobrachium_carcinus.jpg). [file peerj-08-9030-s005.png]
